# Fragmentation of Esophageal Foreign Body With the Holmium: YAG Laser

**DOI:** 10.14309/crj.0000000000000386

**Published:** 2020-05-05

**Authors:** Carolina Mangas-Sanjuan, Lucía Medina-Prado, Sandra Baile-Maxía, Juan Martínez, Juan Antonio Casellas, José Ramón Aparicio

**Affiliations:** 1 Endoscopy Unit, Hospital General Universitario de Alicante, Instituto de Investigación Sanitaria y Biomédica de Alicante (ISABIAL), Alicante, Spain

## CASE REPORT

**Figure 1.**
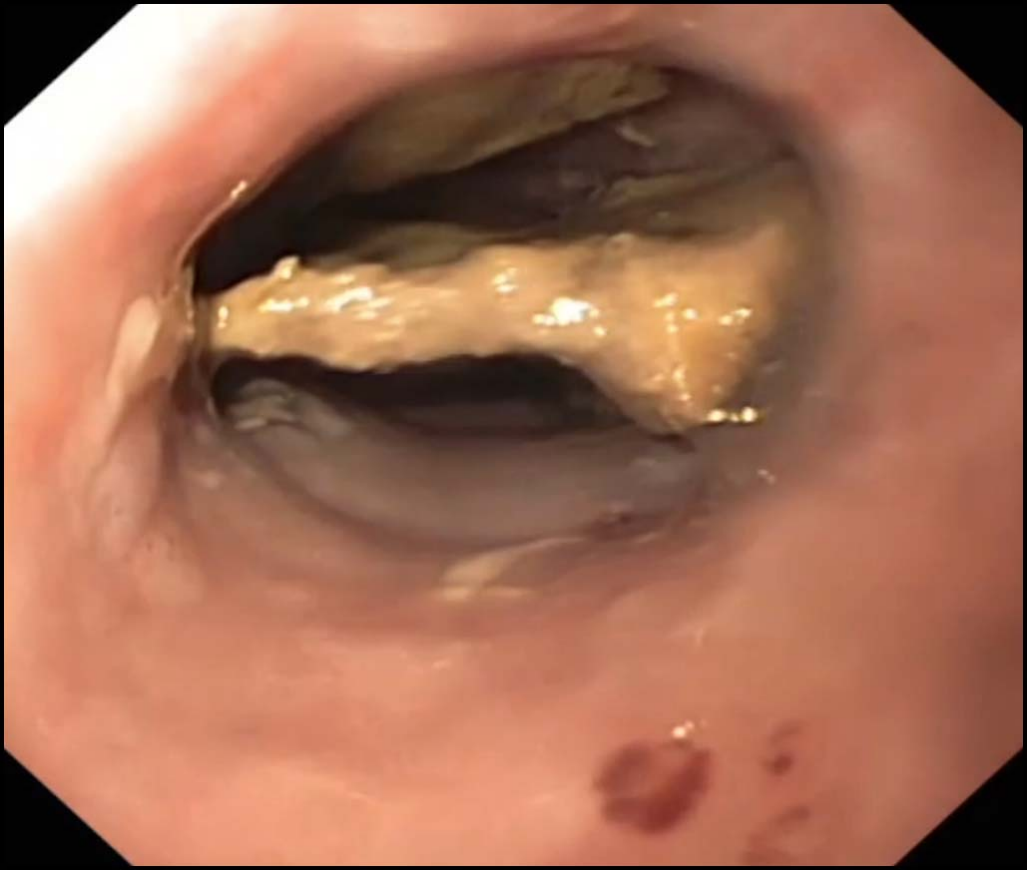
Endoscopic image showing a chicken bone lodged in the distal esophagus.

A 78-year-old man was admitted with hypersalivation and inability to swallow liquids after accidental foreign body ingestion. Esophagogastroduodenoscopy identified a flat and sharp-pointed chicken bone lodged in the distal esophagus (Figure 1). Initial endoscopic removal with a variety of devices (retrieval forceps, polypectomy snares, and even rigid esophagoscopy) was ineffective. An inflated wire-guided balloon dilator was also unsuccessful in retrieving the bone. Computed tomography revealed the absence of esophageal perforation, and 12 hours after admission, the patient was referred to our center.

**Figure 2.**
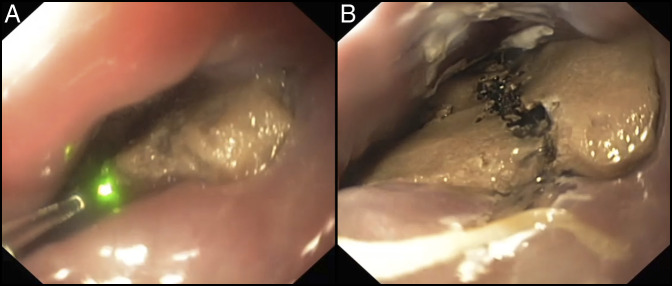
Endoscopy showing (A) the holmium:YAG laser (1.2 J, 10 Hz, 12 W) with a single-use laser fiber (Flexiva 365 laser fiber, Boston Scientific) and (B) the chicken bone fragmented into 2 pieces with the holmium:YAG laser.

**Figure 3.**
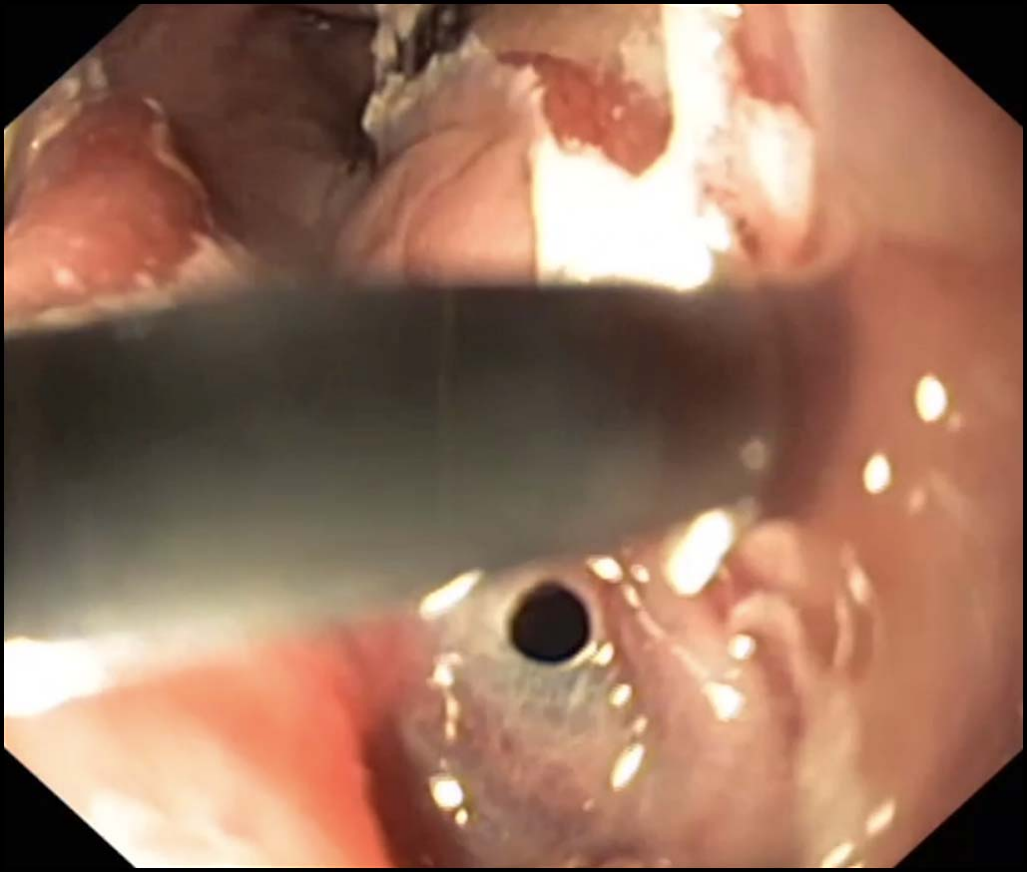
An esophageal perforation of a decubitus ulcer.

Subsequently, esophagogastroduodenoscopy under general anesthesia with endotracheal intubation was carried out. The holmium:YAG laser with a single-use laser fiber (Flexiva 365 μm Laser Fiber; Boston Scientific Co., Natick, MA) and a standard gastroscope (GIF-HQ190, Olympus Medical Systems, Tokyo, Japan) was used to fragment the foreign body into 2 pieces (Video 1, http://links.lww.com/ACGCR/A21 and Figure 2). Pulse energy setting for the laser was 1.2 J energy and 10 Hz frequency. Thereafter, the fragmentation was completed by the use of reusable surgical scissors (FS-3L-1; Olympus Medical System), and the sharp-edged chicken bone pieces were removed using a standard-sized overtube to protect the esophageal mucosa from lacerations. Finally, endoscopic assessment of the esophagus showed a perforation of a decubitus ulcer (<10 mm) (Figure 3). After failed attempt with a standard endoscopic clip, an over-the-scope clip (Ovesco Endoscopy AG, Tübingen, Germany) with pointed teeth (type t) and a cap (11 mm) was deployed. Endoscopic closure of esophageal perforation was successfully achieved and confirmed by radiographic contrast dye injected into the esophagus. The patient was discharged home, asymptomatic after 7 days of hospitalization and antibiotics, without further adverse events.

Esophageal foreign bodies are frequently encountered by endoscopy units. The Holmium:YAG laser is commonly used in urology to treat different pathologies^1^ and has been described in gastrointestinal endoscopic lithotripsy for the treatment of gallbladder stones.^2^ Regarding foreign bodies, few cases of fragmentation within the stomach have been published,^3–5^ and no information about the use of this laser to remove esophageal foreign bodies has been reported. This case demonstrates the safe and successful case report of holmium:YAG laser to fragment difficult esophageal foreign bodies (Video 1, http://links.lww.com/ACGCR/A21).

**Video 1:** Esophagogastroduodenoscopy showing fragmentation of a chicken bone using the holmium:YAG laser.

## DISCLOSURES

Author contributions: C. Mangas-Sanjuan wrote the manuscript and is the article guarantor. L. Medina-Prado, S. Baile-Maxía, J. Martinez, JA Casellas, and JR Aparicio revised the manuscript for intellectual content and approved the final manuscript.

Financial disclosure: J.R. Aparicio is consultant for Boston Scientific.

Previous presentation: This case was presented at the ESGE Days, April 19-21 2018; Budapest, Hungary.

Informed consent was obtained for this case report.
